# Differentiation of Thyroid Nodules (C-TIRADS 4) by Combining Contrast-Enhanced Ultrasound Diagnosis Model With Chinese Thyroid Imaging Reporting and Data System

**DOI:** 10.3389/fonc.2022.840819

**Published:** 2022-06-30

**Authors:** Tiantong Zhu, Jiahui Chen, Zimo Zhou, Xiaofen Ma, Ying Huang

**Affiliations:** ^1^ Department of ultrasound, Shengjing Hospital of China Medical University, Shenyang, China; ^2^ Department of Orthopedics, Shengjing Hospital of China Medical University, Shenyang, China

**Keywords:** Chinese thyroid imaging reporting and data system(C-TIRADS), ultrasound (US), contrast-enhanced ultrasound (CEUS), thyroid nodules, differentiation

## Abstract

**Objectives:**

To establish a contrast-enhanced ultrasound (CEUS) diagnostic schedule by CEUS analysis of thyroid nodules of C-TIRADS 4. To establish a CEUS-TIRADS diagnostic model to differentiate thyroid nodules (C-TIRADS 4) by combining CEUS with Chinese thyroid imaging reporting and data system (C-TIRADS).

**Methods:**

A total of 228 thyroid nodules (C-TIRADS 4) were estimated by CEUS. The arrival time, enhancement degree, enhancement homogeneity, enhancement pattern, enhancement ring, and wash-out time were analyzed in CEUS for all of the nodules. Multivariate factors logistic analysis was performed and a CEUS diagnostic schedule was established. If the nodule had a regular hyper-enhancement ring or got a score of less than 2 in CEUS analysis, CEUS-TIRADS subtracted 1 category. If the nodule got a score of 2 in the CEUS schedule, the CEUS-TIRADS category remained the same as before. If the nodule got a score of more than 2 in the CEUS schedule, CEUS-TIRADS added 1 category. When it reflected an absent enhancement in CEUS, the nodule was judged as CEUS-TIRADS 3. All of the C-TIRADS 4 nodules were re-graded by CEUS-TIRADS. We then compare the diagnosis performance of C-TIRADS, CEUS, and CEUS-TIRADS by sensitivity, specificity, and accuracy.

**Results:**

Among the 228 C-TIRADS 4 nodules, 69 were determined as C-TIRADS 4a, 114 were C-TIRADS 4b, and 45 were C-TIRADS 4c. The sensitivity, specificity, and accuracy of C-TIRADS were 93.1%, 55.3%, and 74.6% respectively. The area under the curve was 0.753. Later arrival time, hypo-enhancement, heterogeneous enhancement, centripetal enhancement, and rapid washout were risk factors of malignancy in multivariate analysis. The sensitivity, specificity, and accuracy of CEUS were 78.7%, 87.5%, and 83.3% respectively. The area under the curve was 0.803. By CEUS-TIRADS diagnostic model combining CEUS with C-TIRADS, a total of 127 cases were determined as malignancy (111 were malignant and 16 were benign) and 101 were diagnosed as benign ones (5 were malignant and 96 were benign). The sensitivity, specificity, and accuracy of CEUS-TIRADS were 95.7%, 85.7%, and 92.1% respectively. The area under the curve was 0.916. The diagnostic performance of CEUS-TIRADS was significantly better than CEUS and C-TIRADS. The difference was statistically significant (P<0.05).

**Conclusions:**

The diagnostic schedule of CEUS could get better diagnostic performance than US in the differentiation of thyroid nodules. The CEUS-TIRADS combining CEUS analysis with C-TIRADS could make up for the deficient sensibility of C-TIRADS, showing a better diagnostic performance than US and CEUS.

## Introduction

Due to the spread of high-resolution ultrasound (US), the detection rate of thyroid nodules has been increased recent in decades. Less than 10% of thyroid nodules are malignant (1, 2). Overdiagnosis and overtreatment have also been the subject of much controversy due to the indolent characteristic of thyroid carcinoma (3, 4). In 2020, Chinese specializations published a Chinese thyroid imaging reporting and data system (C-TIRADS) to diagnose thyroid nodules, which was more convenient and practical in daily clinical practice than the weighting method (5). According to C-TIRADS category, the malignancy risk rate of category 3, 4a, 4b, 4c, and 5 were 0-2%, 2-10%, 10-50%, 50-90%, and more than 90% respectively. Most of the thyroid nodules could be diagnosed correctly by conventional ultrasound alone, especially nodules of category 3 or 5. However, there is diagnostic uncertainty among nodules of C-TIRADS 4. Some benign ones are prone to be diagnosed as malignant, especially nodules of C-TIRADS categories 4a and 4b, which might be recommended to perform a fine needle aspiration (FNA), an invasive form of management (6). In the near past, the strategies for the detection of micro-vessels benefitted by the utility of contrast-enhanced ultrasound (CEUS) were worked out, which proved feasible for the differentiation of benign and malignant thyroid nodules (7–9). Until now, the consensus of the literature about CEUS suggested that later arrival time, hypo-enhancement, heterogeneous enhancement, centripetal enhancement, and earlier wash-out time were the characteristics of malignant nodules. An earlier arrival time, no enhancement or scattered enhancement, iso- or hyper-enhancement, homogeneous enhancement, and regularly peripheral enhancement ring were the features of benign ones (10–12). However, there is no definitive diagnostic criterion of CEUS in differentiating thyroid nodules.

Our study aimed at establishing a CEUS schedule of C-TIRADS 4 thyroid nodules by assigning scores and differentiating uncertain thyroid nodules. At the same time, establishing a CEUS-TIRADS model to increase diagnostic ability by combining CEUS schedule and C-TIRADS. Comparing the diagnostic performance of C-TIRADS, CEUS schedule, and CEUS-TIRADS model.

## Methods

### Study Cohort

**Figure 1:**
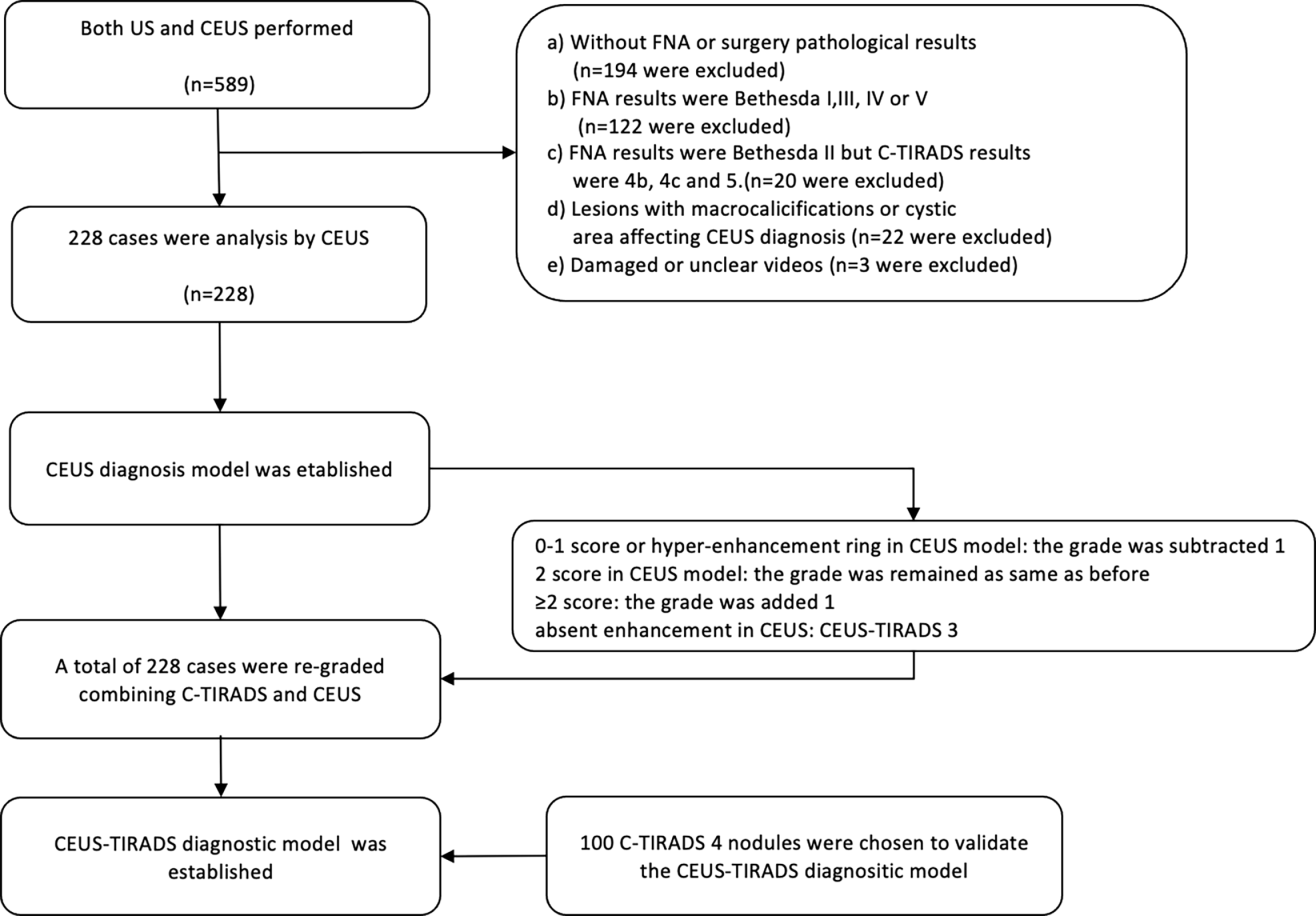
The flow chart of the study. FNA, fine-needle aspiration; US, ultrasound; CEUS, contrast-enhanced ultrasound; C-TIRADS, Chinese imaging reporting and data system.

This study was performed from May 2018 to January 2021, during which 589 C-TIRADS 4 (4a, 4b, 4c) nodules were diagnosed by both US and CEUS. Limited by the sample size of benign cases where pathological results recieved by surgery, this study included several benign cases where pathological results were Bethesda II, established by FNA. To avoid the false-negative results of FNA, the FNA and US results were kept consistent. Inclusive criteria: A) Malignant cases, a) The nodules whose pathological results were malignant got by surgery, c) The nodules revieced pathological results *via* FNA and the Bethesda results were VI category. B) Benign cases, a) The nodules which pathological results were benign got by surgery. b) The pathological results by FNA were Bethesda category II and the results of US were C-TIRADS 4a. Exclusive criteria: a) There were macrocalcifications that affect the CEUS analysis. b) The CEUS images were not entire. c) The results of FNA were Bethesda II but C-TIRADS category was 4b or 4c. d) The results of FNA were Bethesda VI but C-TIRADS category was 4a. In all 318 cases were excluded due to lack of definite pathology results, 20 cases were excluded because of the inconsistency between Bethesda with C-TIRADS results, and 25 were excluded because of the deficiency of images (22 cases with macrocalcifications or cystic area affecting CEUS diagnosis). The US and CEUS images were analyzed by two physicians who had 7-years and 3-years of experience in CEUS separately, being blind to the medical history of the images. Disagreements were resolved by consensus or by the judgment of a third expert who also specialized in CEUS (**Figure 1**).

### CEUS Analysis

The conventional US was performed by an L12-5 transducer (a frequency of 5-12 MHz) of the Philips

IU22 US system (Philips, Bothell, WA, USA). An L9-3 probe, of which the frequency was 7MHz and mechanical index (MI) was 0.07, was facilitated to perform CEUS. Mix 5mL saline with 59mg SonoVue (Bracco, Italy) powder to make a suspension. Extract 1.5mL of the suspension and an intravenous bolus injection was administered manually. CEUS model was chosen to observe dynamically. All of the CEUS procedures were performed by one expert who had 10-years of experience of CEUS. The arrival time (compared with thyroid tissue), enhancement degree (no enhancement, hypo-, iso-, or hyperenhancement), enhancement homogeneity (homogeneity or heterogeneity), enhancement pattern (centripetal or diffuse enhancement), enhancement ring, and wash-out time (compared with thyroid tissue) were analyzed in CEUS for all of the nodules.

### US Analysis

The nodules were graded according to C-TIRADS, and solid, markedly hypoechoic, vertical orientation, microcalcifications, and ill-defined or irregular margin (including extrathyroidal extension) were suspicious malignant signs. Value 1 was added when any aforementioned ultrasound features appeared in the nodule and value 1 was subtracted if the negative feature of a comet-tail artifact was present. A score of 1 wasfor C-TIRADS 4a, 2 for C-TIRADS 4b, and 3-4 for C-TIRADS 4c (5).

### Combining C-TIRADS With CEUS Analysis

All of the C-TIRADS 4 nodules were re-graded by CEUS schedule and CEUS-TIRADS category was got. If the nodule was an absent enhancement in CEUS, the CEUS-TIRADS category was re-determined as 3. If the nodule got a score of 2 in the CEUS schedule, the CEUS-TIRADS category remained the same as before. If the nodule got a score of more than 2 in the CEUS schedule, the CEUS-TIRADS category added 1 based on C-TIRADS. If the nodule had a regular hyper-enhancement ring or got a score of less than 2 in the CEUS schedule, 1 was subtracted from the CEUS-TIRADS category. CEUS-TIRADS 3 and 4a were diagnosed as benignity and CEUS-TIRADS 4b, 4c, and 5 were determined as malignancy. Compare the diagnosis performance of C-TIRADS, CEUS, and CEUS-TIRADS by sensitivity, specificity, and accuracy.

### Prospective Validation of the CEUS-TIRADS Diagnostic Model

To further validate the CEUS-TIRADS diagnostic model, a validation study was performed by a relatively small prospective cohort. A total of 100 C-TIRADS 4 thyroid nodules that were performed on both US and CEUS from February 2021 to October 2021 were chosen. The inclusive and exclusive criteria were the same as the study cohort. The sensitivity, specificity, accuracy, and ROC of C-TIRADS, CEUS, and CEUS-TIRADS were calculated.

### Statistical Analysis

SPSS software (version 26.0, IBM Corporation, Armonk, NY, USA) was used for statistical analysis. Quantitative data are expressed as the mean value ± standard deviation ( $\bar{X}$ ± standard deviation). Two independent samples t-test was used to describe the differences in age and size between benign and malignant nodules. The differences in CEUS parameters between benign and malignant nodules were analyzed by χ^2^-test. Multivariate factors logistic analysis was performed to establish a CEUS diagnosis model. The receiver operating characteristic (ROC) curves obtained by SPSS software were used to calculate the optimal cut-off points. The area under the curve (AUC) was used to compare the diagnostic performance.

## Results

### Basic Characteristics

A total of 228 thyroid nodules (C-TIRADS 4) were included. Among them, 116 were malignant and 112 were benign. When 134 patients recieved pathological results by surgery, 79 were malignant (74 were papillary thyroid carcinoma, 3 were follicular thyroid carcinoma, 1 was lymphoma and 1 was medullary carcinoma), and 55 were benign (48 were nodular goiter and 7 were Hashimoto’s thyroiditis). A further 94 patients obtained the pathological results by FNA (57 were Bethesda category II and 37 were Bethesda category VI). The age of patients was difference significantly between benign and malignant groups. There are no significant differences in sex and size between the two groups. (**Table 1**)

**Table 1:** Basic characteristics of the included nodules (n=228).

	Malignant (n=116)	Benign (n=112)	*P* value
Sex			0.657
male	23	20	
female	93	92	
Age (years)	41.6±11.1	46.9±12.7	0.001*
Size (mm)	9.2±5.8	11.1±8.1	0.077

*P Value < 0.05 indicates statistical significance.

### US Analysis

Among the 228 nodules, 69 were determined as C-TIRADS 4a (62 were benign and 7 were malignant), 114 were C-TIRADS 4b (47 were benign and 67 were malignant) and 45 were C-TIRADS 4c (4 were benign and 41 were malignant). The sensitivity, specificity, and accuracy of CEUS were 93.1% (108/116), 55.3% (62/112), and 74.6% (170/228) respectively. The AUC was 0.753.

### CEUS Analysis

Twenty nodules were not enhancemed in CEUS. The CEUS parameter was compared between benign and malignant nodules among 208 nodules. Arrival time, enhancement degree, enhancement homogeneity, and enhancement pattern peripheral enhancement ring were statistically different between benign and malignant nodules (*P*<0.05). There are no significant differences in sex and size between the two groups. Later arrival time, hypo-enhancement, heterogeneous enhancement, and centripetal enhancement were the characteristics of malignant nodules. Earlier arrival time, no enhancement, iso-enhancement or hyper-enhancement, homogeneous enhancement, diffusion enhancement, and peripheral hyper-enhancement ring were the features of benign nodules. Later arrival time, hypo-enhancement, heterogeneous enhancement, and centripetal enhancement were risk factors in multivariate analysis. The scores of every aforementioned factor being got by multivariate analysis were 24, 39, 6, and 12 respectively. In the counting method, the weighting values based on the ORs of the four features were as follows: 2 for hypo-enhancement, 1 for later arrival time, heterogeneous enhancement, and centripetal enhancement respectively. (**Table 2**) Nodules with scores of greater than or equal to 2 were determined as a malignant ones, according to a cutoff value calculated by the ROC curve. Nodules showing no enhancement, peripheral hyper-enhancement ring, and a score of 0-1 in the CEUS diagnosis model were diagnosed as benign. A total of 100 nodules (85 malignant and 15 benign) were diagnosed as malignant in the CEUS schedule, While 128 nodules (97 benign and 31 malignant) were determined as benign. The sensitivity, specificity, and accuracy of CEUS were 73.3% (85/116), 86.6% (97/112), and 80.9% (182/225) respectively. The AUC was 0.803.

**Table 2:** CEUS characteristics between malignant and benign thyroid nodules (n=208).

	Malignant(n=116)	Benign(n=92)	χ^2^ value	*P* value
Sex				0.994
male	23	16		
female	92	76		
Age (years)	41.6±11.2	46.4±13.2		0.006*
Size (mm)	9.2±5.8	11.7±10.3		0.069
Arrival time			24.333	0.000*
Later arrival time	52	12		
Equal or earlier arrival time	64	80		
Enhancement degree			39.208	0.000*
Hypo-enhancement	73	18		
Iso-enhancement	26	45		
Hyper-enhancement	17	29		
Enhancement homogeneity			5.998	0.014*
Homogeneity	42	73		
Heterogeneity	74	19		
Enhancement pattern			11.644	0.001*
Centripetal	88	6		
Diffuse	28	86		
Wash-out Time			3.216	0.073
Earlier	20	8		
Later or equal	96	84		
Peripheral enhancement ring			40.796	0.000*
Yes	1	28		
No	115	64		

*P Value <0.05 indicates statistical significance.

### The Establishing of CEUS-TIRADS Diagnostic Model by Combining C-TIRADS With CEUS Analysis

According to the CEUS diagnosis schedule, all of the C-TIRADS 4 nodules were re-graded. By CEUS-TIRADS diagnostic model combining CEUS with C-TIRADS, a total of 127 cases were determined as malignant (111 were malignant and 16 were benign) and 101 were diagnosed as benign ones (5 were malignant and 96 were benign). Among 69 C-TIRADS 4a nodules, 57 were re-determined as CEUS-TIRADS 3, 10 were re-determined as CEUS-TIRADS 4b, and 2 remained CEUS-TIRADS 4a. Among 114 nodules for C-TIRADS 4b, 12 were re-graded as CEUS-TIRADS 3 (CEUS was no enhancement). Meanwhile 29 were re-graded as CEUS-TIRADS 4a, 25 were maintained as CEUS-TIRADS 4b as before, and 48 were re-graded as CEUS-TIRADS 4c. Among the 45 nodules for C-TIRADS 4c, 1 was re-defined as CEUS-TIRADS 3, 29 were re-defined as CEUS-TIRADS 4b, 5 were CEUS-TIRADS 4c and 10 were re-defined as CEUS-TIRADS 5. (**Table 3**)

**Table 3:** The accurate diagnosis cases of C-TIRADS, CEUS and CEUS-TIRADS diagnostic model.

		Pathology	
		Malignant (n=116)	Benign (n=112)
C-TIRADS	Malignant (4b and 4c)	108	51
	Benign (4a)	7	62
CEUS	Malignant (score≥2)	85	15
	Benign (score<2)	31	97
CEUS-TIRADS	Malignant (4b,4c or 5)	111	16
	Benign (3 or 4)	5	96

**Figure 2:**
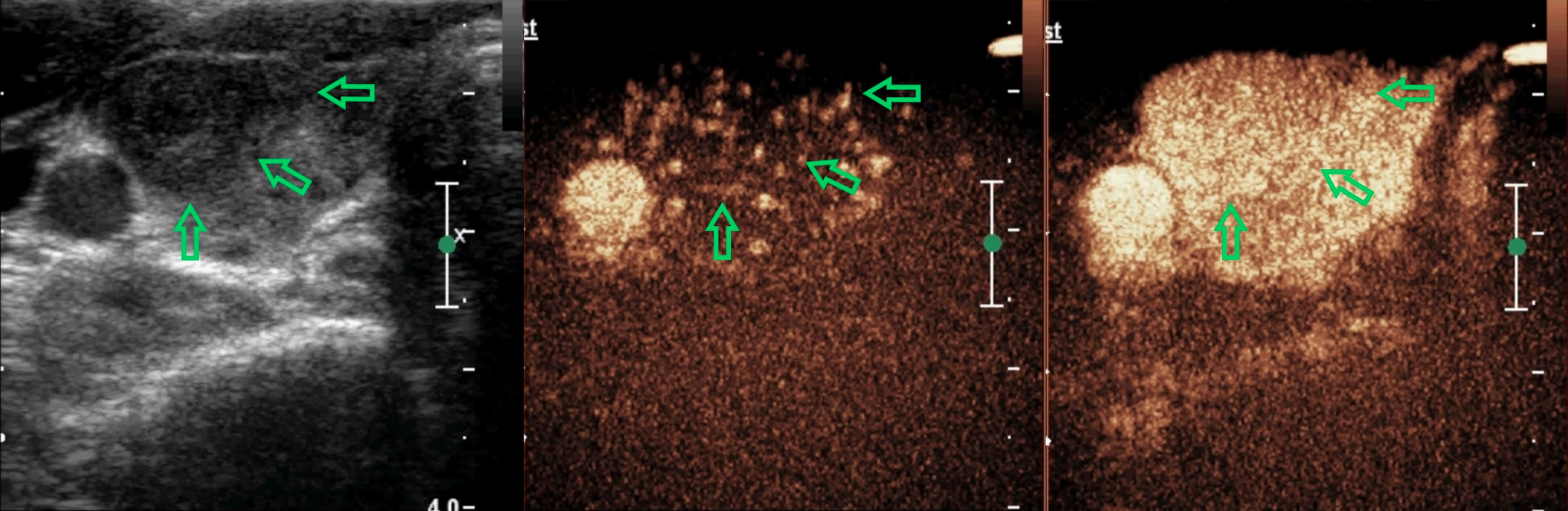
A 38-year-old woman with a nodule in the right-lobe of her thyroid gland. There are two suspicious signs with the nodule (solid and irregular margin) and it was defined as C-TIRADS 4b. In CEUS analysis, it reflected as equal arrival time, iso-enhancement, homogeneity, and diffuse enhancement, receiving a score of 0 in the CEUS model. The CEUS-TIRADS category was 4a. The pathological result was Hashimoto’s thyroiditis.

**Figure 3:**
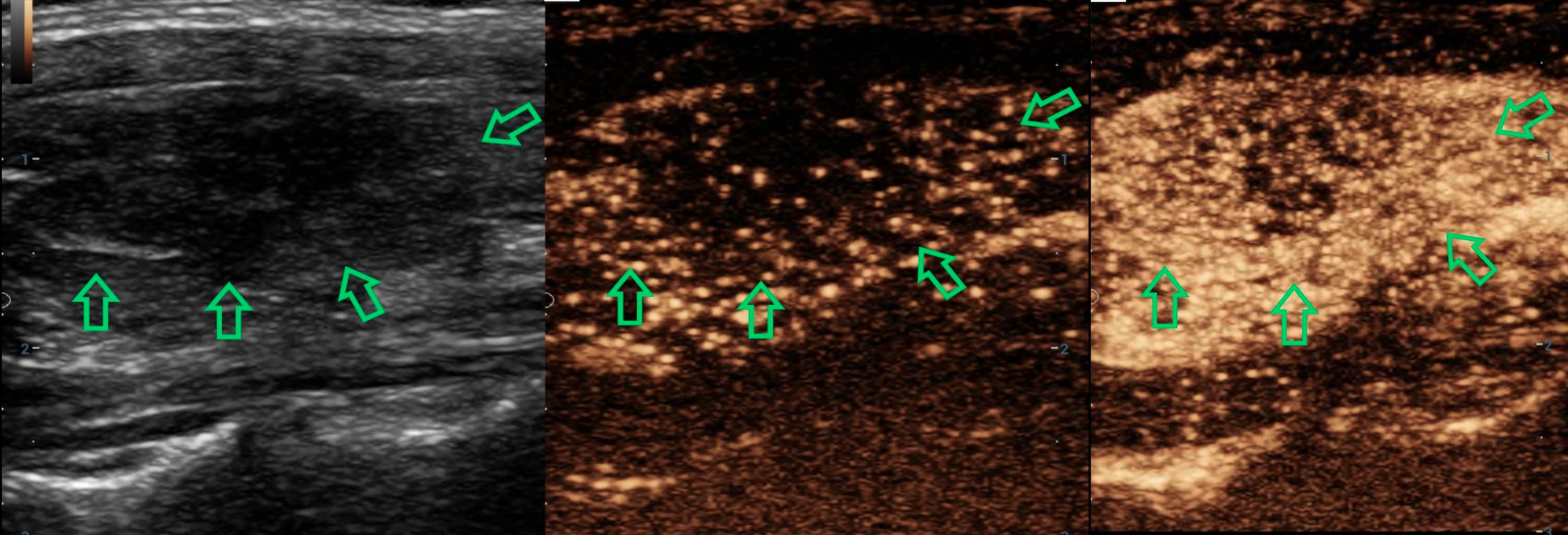
A 35-year-old woman with a nodule in the left-lobe of her thyroid gland. There are two suspicious signs with the nodule (solid and irregular margin) and it was defined as C-TIRADS 4b. In CEUS analysis, it reflected as later arrival time, hypo-enhancement, heterogeneous and centripetal enhancement, getting a score of 4 in the CEUS model. The CEUS-TIRADS category was 4c. The pathological result was papillary thyroid carcinoma.

The final results by combining CEUS and C-TIRADS were changed in 52 nodules after re-grading. The results of 45 nodules were amended by CEUS correctly. Among them, 40 benign cases were misdiagnosed as C-TIRADS 4b or 4c but corrected to CEUS-TIRADS 3 or 4a after being regraded by the CEUS-TIRADS diagnostic model. Among the 40 benign nodules, 13 nodules of C-TIRADS 4b or 4c were re-determined as CEUS-TIRADS 3 resulting from no enhancement in CEUS. However, 27 C-TIRADS 4b nodules were degenerated as CEUS-TIRADS 4a because of receiving a score of less than 2 or peripheral hyper-enhancement ring in CEUS analysis. The false-positive results of C-TIRADS were corrected. Five C-TIRADS 4a nodules were upgraded as CEUS-TIRADS 4b due to getting a score of more than 2 in CEUS, with a modification for the false-negative results of C-TIRADS. There are 7 nodules giving an incorrect result after re-grading. Five benign ones for C-TIRADS 4a scored more than 2 in CEUS (false positivity), with a wrong upgraded category. Two malignant ones for C-TIRADS 4b scored less than 2 or peripheral hyper-enhancement ring in CEUS analysis (false negatively) and the CEUS-TIRADS category was transformed to 4a. (**Figure 2**, **Figure 3**)

**Figure 4:**
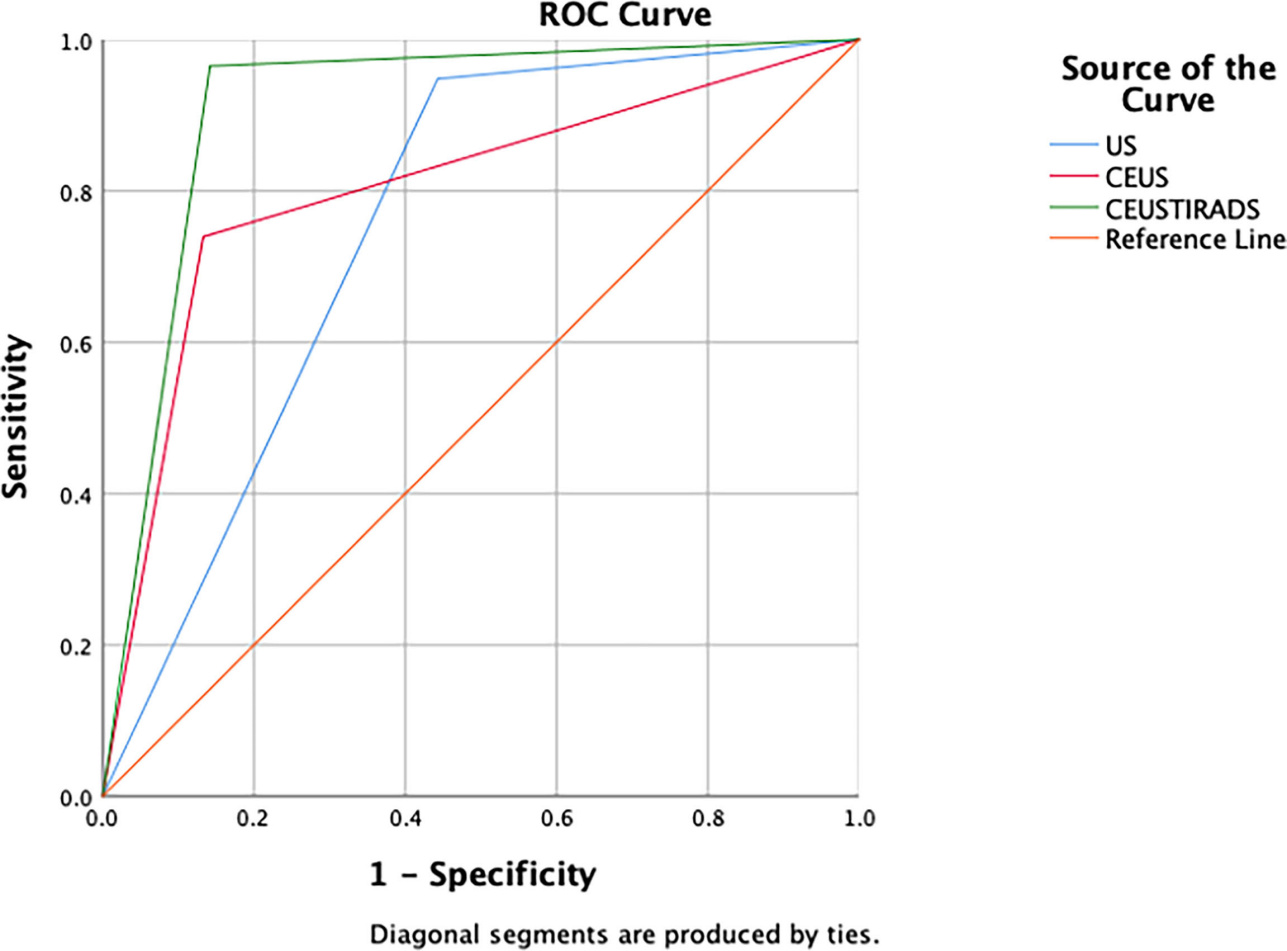
The ROC curves of C-TIRADS, CEUS, and CEUS-TIRADS of 228 nodules in the diagnostic model.

In general, the sensitivity, specificity, and accuracy of combining C-TIRADS with CEUS were 95.7% (111/116), 85.7% (96/112), and 92.1% (207/228) respectively. The AUC was 0.916. The diagnostic performance of combining C-TIRADS and CEUS was significantly better than CEUS and C-TIRADS. The difference was statistically significant (*P*<0.05). (**Figure 4**) (**Table 4**)

**Table 4:** Diagnosis performance of C-TIRADS, CEUS and CEUS-TIRADS diagnostic model.

	Sensitivity (%)	Specificity (%)	Accuracy (%)	AUC	Asymptotic 95% confidence interval		*P* value
					Lower Bound	Upper Bound	
C-TIRADS	93.1	55.3	74.6	0.753	0.688	0.818	0.000*
CEUS	73.3	86.6	80.9	0.803	0.744	0.863	0.000*
CEUS-TIRADS	95.7	85.7	92.1	0.916	0.874	0.958	0.000*

*P Value <0.05 indicates statistical significance.

**Figure 5:**
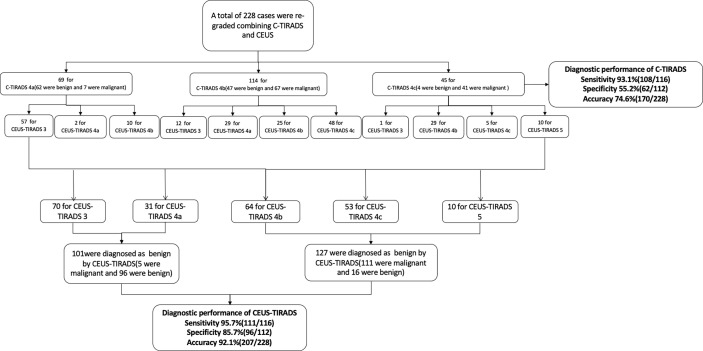
The process of establishing of CEUS-TIRADS model.

The process of establishing of CEUS-TIRADS model was shown in **Figure 5**.

### Prospective Validation of the CEUS-TIRADS Diagnostic Model

**Figure 6:**
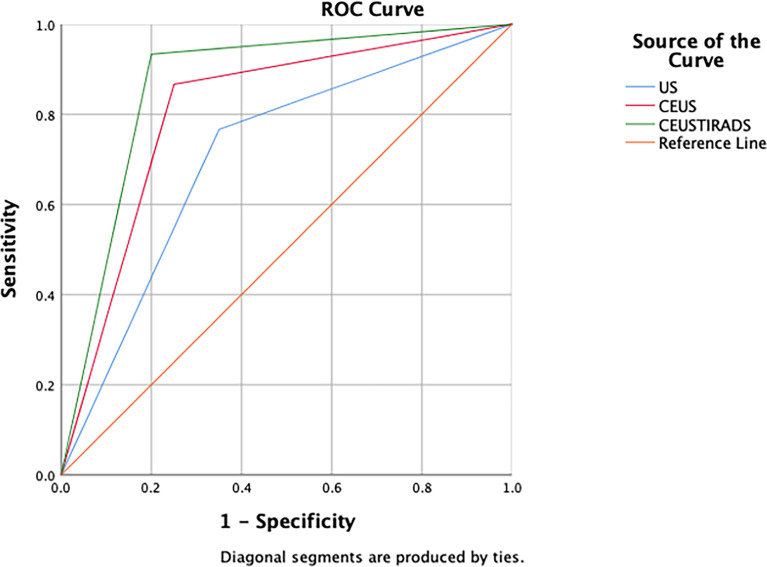
The ROC curves of C-TIRADS, CEUS, and CEUS-TIRADS of 100 nodules in the validation cohort.

In 100 cases of validation cohort, 61 were malignant and 39 were benign. The sensitivity, specificity, and accuracy of C-TIRADS were 73.7% (45/61), 66.7% (26/39), and 71% (71/100) respectively. The sensitivity, specificity, and accuracy of CEUS were 85.2% (52/61), 74.4% (29/39), and 81% (81/100) respectively. The sensitivity, specificity, and accuracy of in CEUS-TIRADS diagnostic model were 95.1% (58/61), 79.5% (31/39), and 89% (89/100) respectively. The AUC of C-TIRADS, CEUS, and CEUS-TIRADS were 0.698, 0.798, and 0.873 respectively (*P*<0.05). (**Figure 6**) (**Table 5**)

**Table 5:** Diagnosis performance of C-TIRADS, CEUS and CEUS-TIRADS diagnostic model in validation cohort.

	Sensitivity (%)	Specificity (%)	Accuracy (%)	AUC	Asymptotic 95% confidence interval		*P* value
					Lower Bound	Upper Bound	
C-TIRADS	73.7	66.7	71	0.698	0.589	0.816	0.000*
CEUS	85.2	74.4	81	0.798	0.702	0.894	0.000*
CEUS-TIRADS	95.1	79.5	89	0.873	0.791	0.955	0.000*

*P Value <0.05 indicates statistical significance.

**Figure 7:**
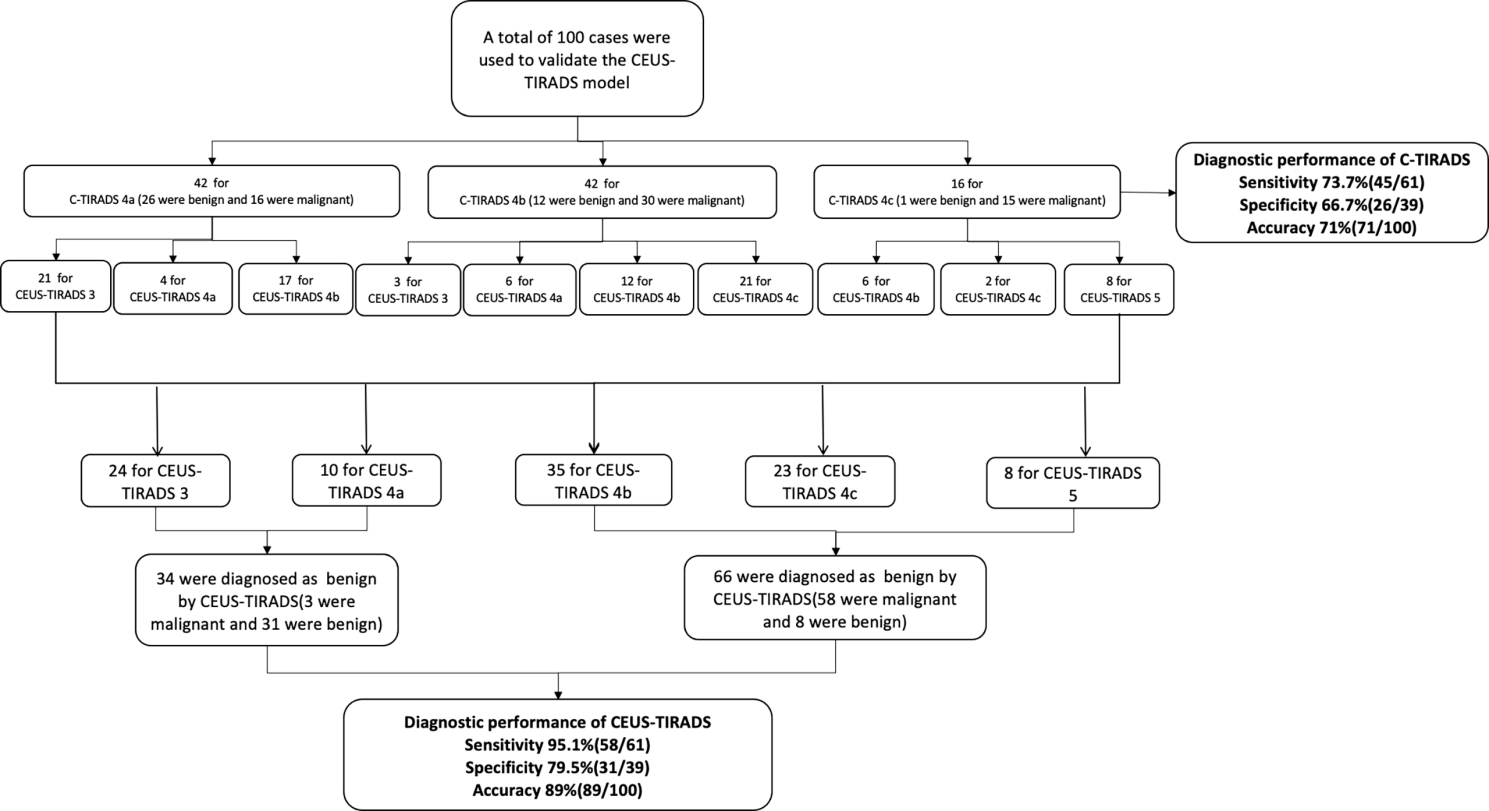
The process of validation of CEUS-TIRADS model.

The process of validation of the CEUS-TIRADS model was shown in **Figure 7**.

## Discussion

Ultrasound is the most frequently used imaging modality in diagnosing thyroid and parathyroid lesions (1, 6, 13). For the nodules of category 4, some guidelines suggested an FNA to make a further diagnosis (6, 14). The malignancy rate of C-TIRADS 4a and 4b were 2-10% and 10-50% respectively. (5) There is a considerable number of benign nodules that could not be recognized by conventional US. To increase the diagnosis ability of US in nodules of C-TIRADS 4, our study chose these uncertain cases to take further analysis. The sensitivity, specificity, and accuracy of C-TIRADS were 93.1%, 55.3%, and 74.6% respectively. The area under the curve was 0.753, which was lower than the published data of 0.890 due to the differences in study objects possibly.

In recent years, more and more studies aimed at improving the diagnostic performance of thyroid nodules by multiple US models (15). CEUS takes important part in the differentiation of thyroid nodules, the assistance of FNA, and the estimation of therapeutic effect after radiofrequency ablation (RFA) (11, 16). In most of the diagnostic studies, the different CEUS characteristics between benign and malignant nodules were compared, but the diagnostic criterion of CEUS in differentiation was not available yet. Zhu et al. chose six suspicious signs as positive indexes, and the optimal threshold score of CEUS was 3 (AUC=0.884) (17). In their study, each of the positive manifestations was assigned 1 point but not calculated by ORs values as in our study. In our study, wash-out time was excluded in the multivariance analysis. The hypo-enhancement took the most important place (OR value of 39) and was valued as 2 in the diagnosis model. The importance of other factors decreased progressively as followed: later arrival time (OR of 24), centripetal enhancement (OR of 12), and heterogeneous enhancement (OR of 6). One point of score was added for each of them. The assigned score of every factor was simple and convenient for clinical practice. No enhancement and peripheral hyperenhancement ring were specific indexes that indicated benignity. The diagnostic performance of the CEUS model was better than C-TIRADS (*P*<0.05), with sensitivity, specificity, and accuracy of 84.8%, 76.0%, and 79.9% respectively (AUC=0.803).

Several studies aimed at differentiating thyroid nodules combining CEUS and US, getting a better diagnostic result. Liu et al. studied 102 thyroid nodules (≤10mm) of category 3 or 4 in Korean TIRADS (18), and logistic regression analysis of US combined with CEUS demonstrated that taller than wide, microcalcification, suspicious lymph gland, slow enhancement time, and absence of rim-like enhancement are risk factors of malignancy. Conventional US combined with CEUS had a superior diagnostic performance for TI-RADS 3 and 4 thyroid micronodules compared with conventional US and CEUS alone. Zhang et al. improved TIRADS and combined it with CEUS, increasing the diagnostic accuracy, particularly in lesions with a TIRADS score of 4a and 4b (19).

In our study, 45 misdiagnosed nodules based on C-TIRADS alone were corrected by CEUS. Among them, 13 nodules were category 4b or 4c in C-TIRADS but showed no enhancement in CEUS, getting a final result of category 3 (FNA results were Bethesda II). It was found that 27 C-TIRADS 4b nodules scored less than 2 in CEUS, and the category was returned as 4a which is consistent with pathological results. Five malignant nodules of C-TIRADS 4a were diagnosed correctly by CEUS. However, 27 misdiagnosed nodules based only on CEUS were corrected by C-TIRADS (C-TIRADS 4c), and the false-negative results of CEUS did not affect the final diagnosis. The higher sensitivity of C-TIRADS and higher specificity of CEUS could each make up for the deficiency of the other, leading to an improvement in diagnostic performance.

There are 8 nodules being misdiagnosed by CEUS that were originally diagnosed correctly by C-TIRADS. The size of the 5 benign ones (2 were Hashimoto’s thyroiditis and 3 were nodular goiter) and the 3 malignant ones (both of them were PTC) were all less than 10mm. According to the studies of Wu (10) and Zhao (20), CEUS may get a lower diagnostic accuracy in nodules less than 10mm. How to improve the diagnostic performance in nodules less than 10mm would be a future direction for our research.

In total, there were 21 cases that failed to be diagnosed in the CEUS-TIRADS diagnostic model, 5 were malignant and 16 were benign. In the 5 malignant cases (all of them were PTC), 3 nodules for C-TIRADS 4b recieved a score of 0-1 in CEUS. This is the falsely negative result of CEUS leading to misdiagnosis. Two nodules for C-TIRADS 4a received a score of 1 in CEUS. Both C-TIRADS and CEUS failed to diagnose the nodule. PTC could be classified into three major subtypes according to the biological behavior: subtypes associated with aggressive outcomes, subtypes associated with less favorable outcomes, and subtypes associated with favorable outcomes (21). From the previous studies, there were no obvious differences among various subtypes in ultrasonographic features (22, 23). However, the CEUS characteristics among different subtypes had not been reported yet. It was speculated that the pathological subtypes of PTC affected the CEUS results, and future related studies should be put forward.

Among 16 benign cases that failed to be diagnosed in the CEUS-TIRADS diagnostic model, 6 were Hashimoto’s thyroiditis, 9 were nodular goiter, and 1 was nodular goiter with fibrosis. Two nodules were C-TIRADS 4c and got a score of 1 in CEUS, the change of category did not make any difference to the final results. Nine nodules failed to be diagnosed by both CEUS and C-TIRADS. Five nodules for C-TIRADS 4a were misdiagnosed in CEUS. Yang et al. suggested (24), that in the background of Hashimoto’s thyroiditis, the heterogenicity and hypervascularity of the thyroid parenchyma may change the relative enhancement discrepancy between background with lesions. The different stages of nodular goiters, such as cystic degeneration, fibrosis, and calcification, may be the influence factors of different CEUS manifestations.

From our study, the C-TIRADS had a lower specificity and the CEUS had a lower sensitivity. Combining C-TIRADS and CEUS could make them complementary of each other, granting a better diagnostic ability. The sensitivity, specificity, and accuracy were 95.7%, 85.7%, and 92.1% respectively (AUC=0.916).

In several previous studies, the conventional US features and CEUS characteristics were taken together to be analyzed. Some suspected US and CEUS characteristics may be excluded in multivariance analysis (25, 26). But in clinical practice, being limited by technology or acceptance of patients, CEUS has not been extensively promoted at present. From our perspective, to be more practical and reasonable during daily clinical work, CEUS analysis should base on the diagnosis results of conventional US. The cases which scored 2 in CEUS were maintained as the same category as before, which could avoid false-positive results of CEUS as much as possible. In our study, CEUS acted as an assisted diagnosis modality by modulating the C-TIRADS category, which could maximize the diagnostic process. With a convenient counting method, the diagnostic model could be applied in clinical practice easily.

### Limitations

This study has several limitations. First, all of the cases in establishing the CEUS schedule were C-TIRADS 4 nodules which may lead to a selection bias. More different category cases should be estimated further to detail the classification and weighting method in future studies. Second, this study is retrospective research. To avoid information bias, we use a prospective cohort to validate CEUS-TIRADS model. The population size of the prospective cohort is small, and a larger patient population from more centers should be involved to further verify the CEUS-TIRADS model. Third, limited by the population size of surgery benign cases, we included a certain amount of Bethesda II nodules that pathological results got from FNA. To avoid the false negative results of FNA, our study excluded the cases of inconsistency between FNA and US. However, there may be sporadic cases being misdiagnosed by FNA. More benign cases with surgery pathological results should be included in the future.

This study established a TIRADS-CEUS diagnostic model to improve the diagnostic performance of C-TRIADS 4 thyroid nodules by combing US and CEUS. More uncertain C-TRIRADS 4 nodules will get confirmed diagnosis with the help of TIRADS-CEUS model, which could avoid FNA, especially for benign nodules confirmed by this model. For Bethesda I or III or IV nodules, this model could enhance the diagnostic confidence and improve the diagnosis and treatment process.

## Conclusions

The diagnostic model of CEUS could give better diagnostic performance than US in the differentiation of thyroid nodules. The CEUS-TIRADS combining CEUS analysis with C-TIRADS could make up for the deficient sensibility of C-TIRADS, showing a better diagnostic performance than US and CEUS.

## Data Availability Statement

The original contributions presented in the study are included in the article/supplementary material. Further inquiries can be directed to the corresponding author.

## Ethics Statement

The studies involving human participants were reviewed and approved by Shengjing Hospital of China Medical University. The patients/participants provided their written informed consent to participate in this study.

## Author Contributions

TZ had the conception and design of this study. TZ and YH provided the study materials and patients. JC and XM analyzed the images and videos. ZZ performed the statistical analysis. TZ wrote the manuscript. All of the authors read and approved the final manuscript.

## Funding

This study was supported by grants from the health care big data research project of China Medical University (HMB201902103)and 345 Talent Project. YH is the recipient of the funding.

## Conflict of Interest

The authors declare that the research was conducted in the absence of any commercial or financial relationships that could be construed as a potential conflict of interest.

## Publisher’s Note

All claims expressed in this article are solely those of the authors and do not necessarily represent those of their affiliated organizations, or those of the publisher, the editors and the reviewers. Any product that may be evaluated in this article, or claim that may be made by its manufacturer, is not guaranteed or endorsed by the publisher.
